# tDCS over the Left Prefrontal Cortex Enhances Cognitive Control for Positive Affective Stimuli

**DOI:** 10.1371/journal.pone.0062219

**Published:** 2013-05-21

**Authors:** Marie-Anne Vanderhasselt, Rudi De Raedt, Andre R. Brunoni, Camila Campanhã, Chris Baeken, Jonathan Remue, Paulo S. Boggio

**Affiliations:** 1 Department of Experimental Clinical and Health Psychology, Ghent University, Ghent, Belgium; 2 Clinical Research Centre, University Hospital, University of São Paulo, São Paulo, Brazil; 3 Department of Neurosciences and Behaviour, Institute of Psychology, University of São Paulo, São Paulo, Brazil; 4 Social and Cognitive Neuroscience Laboratory and Developmental Disorders Program, Centre for Health and Biological Sciences, Mackenzie Presbyterian University, São Paulo, Brazil; 5 Department of Psychiatry and Medical Psychology, Ghent University, Ghent, Belgium; 6 University and Department of Psychiatry, University Hospital UZBrussel, Brussels, Belgium; Institute of Psychology, Chinese Academy of Sciences, China

## Abstract

Transcranial Direct Current Stimulation (tDCS) is a neuromodulation technique with promising results for enhancing cognitive information processes. So far, however, research has mainly focused on the effects of tDCS on cognitive control operations for non-emotional material. Therefore, our aim was to investigate the effects on cognitive control considering negative versus positive material. For this sham-controlled, within-subjects study, we selected a homogeneous sample of twenty-five healthy participants. By using behavioral measures and event related potentials (ERP) as indexes, we aimed to investigate whether a single session of anodal tDCS of the left dorsolateral prefrontal cortex (DLPFC) would have specific effects in enhancing cognitive control for positive and negative valenced stimuli. After tDCS over the left DLPFC (and not sham control stimulation), we observed more negative N450 amplitudes along with faster reaction times when inhibiting a habitual response to happy compared to sad facial expressions. Gender did not influence the effects of tDCS on cognitive control for emotional information. In line with the Valence Theory of side-lateralized activity, this stimulation protocol might have led to a left dominant (relative to right) prefrontal cortical activity, resulting in augmented cognitive control specifically for positive relative to negative stimuli. To verify that tDCS induces effects that are in line with all aspects of the well known Valence Theory, future research should investigate the effects of tDCS over the left vs. right DLPFC on cognitive control for emotional information.

## Introduction

Transcranial Direct Current Stimulation (tDCS) is a relatively new neuromodulation technique that consists in applying a direct electric current through electrodes positioned over one's scalp, inducing polarity-dependent effects that last beyond the period of stimulation (for a review, see [1]). Although there exist various potential stimulation spots [1], the dorsolateral prefrontal cortex (DLPFC) is often targeted when exploring tDCS effects on cognition [2], [3], [4]. Nevertheless, until now research has mainly focused on the effects of tDCS on cognitive control operations for non-emotional material (for a review, see [5]), even though imaging studies have also associated the DLPFC with cognitive control for emotional material [6]. There is a clear need for studies looking at the effects of tDCS on cognitive control over emotional information. In an attempt to fill this gap, a recent study of [7] looked at the effects of tDCS on cognitive control for emotional information in healthy volunteers and depressed patients. Although these authors found that a single session of tDCS over the left DLPFC ameliorated cognitive control for emotional information in healthy volunteers, they did not differentiate between positive and negative valenced material. Therefore, the aim of the present study in healthy volunteers was to specifically look into the effects of tDCS applied to the DLPFC on cognitive control for emotionally positive and negative material.

**Figure 1:**
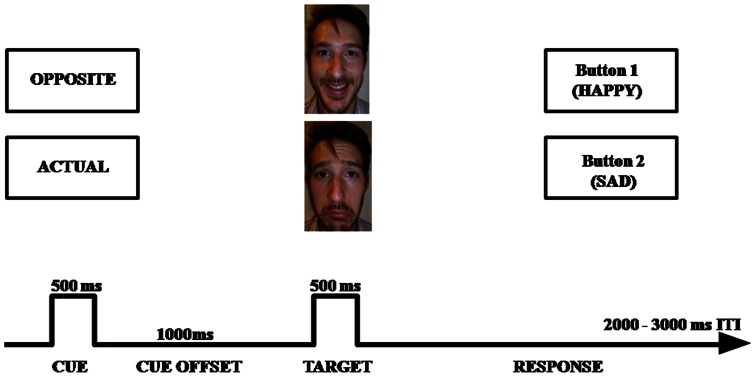
Schematic overview of the Cued Emotional Conflict Task (CECT). First, a cue is presented in the center of the screen (“actual” or “opposite”), followed by a face with an emotional expression (happy or sad). The face shown in the illustration is not from the KDEF database. The individual of the photograph has given written informed consent, as outlined in the PLOS consent form, to publication of his photograph.

Cognitive control refers to the ability to change one's behavior in the pursuit of current goals and context representations [8]. In the current paper, cognitive control for emotional information is evaluated using the Cued Emotional Control Task (CECT), see (Figure 1). In this task, a precedent cue instructs participants to either respond to the actual or opposite emotion of a facial expression subsequently displayed. The need for cognitive control is higher during the latter condition, as participants must overcome a habitual response in order to engage towards the opposite emotion. Not only reaction times (RT) are longer in this condition, but also event related potentials (ERPs) show an enhanced frontal-central negative voltage deflection that peaks between 400–500 ms [9], which is assumed to index conflict monitoring and interference resolution [10], [11]. It is important to point out that, based on the CECT cue-target design, it is possible to disentangle stimulus identity (i.e., valence of the face) and conflict (i.e., actual or opposite cue), which makes it possible to investigate information processing for particular affective material.

All together, because the majority of studies so far have looked at the effects of tDCS on cognitive control for non-emotional information, we aimed to investigate whether anodal tDCS of the left DLPFC would have specific effects in enhancing cognitive control for emotional information (positively versus negatively valenced), using behavioral and ERP data as indexes. ERP correlates make it possible to investigate neural mechanisms underlying cognitive control processes, and whether they are differently influenced by tDCS for positive or negative material. Left sided stimulation was chosen because most studies have observed enhanced cognitive control (for non-emotional information) after tDCS of the left DLPFC [2], [3], [12], [13]. Further, a study of [14] observed enhanced response inhibition (i.e., number of errors on a go/nogo task) for positive material following tDCS of the left DLPFC in a group of 26 major depressed patients [14]. Based on this prior (behavioral) neuromodulation study of [14], our tentative hypothesis was that a single, active tDCS session in healthy volunteers (using the same montage as [14]) would be associated with faster RTs and enhanced (more negative) N450 amplitudes specifically when inhibiting a habitual response towards positive compared to negative stimuli. For the sham condition, we expected no changes in RTs or ERP amplitudes. Finally, in order to exclude the possibility that mood changes would influence our results, mood states were also assessed.

## Methods

The study was conducted in adherence to the Declaration of Helsinki and was approved by the institutional ethics committee of the Mackenzie Presbyterian University, Brazil and by the National Ethics Committee (SISNEP, Brazil).

### Participants

Twenty-five (8M/17F) healthy volunteers with a mean age of 22.12 years (*SD*  = 3.76) participated in this study. Exclusion criteria were: (1) current or past psychiatric or neurological disorders (including cerebral concussion); (2) substance abuse in the last year; (3) lifetime substance dependence; (4) current psychotropic medication. All participants were right handed and had normal or corrected to normal vision.

### Procedure

Written informed consent was obtained from all participants. A sham (placebo)-controlled crossover design was used; participants received 20 minutes of active and sham stimulation. The order of both stimulation sessions (real tDCS and sham stimulation) was counterbalanced, with an interval of at least 48 hours. Approximately 15 minutes following the end of the stimulation (the time needed for the placement of the EEG net), participants performed the CECT during which EEG was recorded. In addition, subjective mood ratings were recorded using the Positive Affect and Negative Affect [15] scale (PANAS) at three time points: baseline (T_0_), immediately after stimulation (T_1_), and after task performance (approximately 60 minutes after stimulation, T_2_).

### Material and apparatus

#### CECT

The CECT was programmed using E-prime (Psychology Software Tools Inc, Pittsburgh, Pennsylvania). Each trial started with one of two word cues presented for 500 ms, see (Figure 1): “actual”, which instructed participants to press a key corresponding to the emotional expression of the upcoming target face (e.g., press “happy” when a happy face is presented); and “opposite”, which indicated that participants should make the response corresponding to the opposite emotional expression of the target face (e.g., press “happy” when a sad face is presented). Following the cue word, a black screen was presented for 1500 ms. After this fixed inter-stimulus interval, either a happy or sad face was presented until participants responded. Eighteen faces (9F/9M) from the Karolinska Directed Emotional Faces dataset [16] were used. Each of these 18 faces was shown in a happy or sad expression. Importantly, the sample of positive and negative valenced pictures were matched for arousal with an overall average of about 4 on a 1 (calm) to 9 (aroused) point scale [17]. Faces were selected if normative ratings indicated that more than 75% of the raters categorized the facial expression correctly with an average intensity rating higher than 6 on a 1 (not intense) to 9 point scale (intense) [17]. Participants were instructed to respond as quickly and accurately as possible immediately after the face presentation; the assignment of labels (happy or sad) to the two buttons was counterbalanced across participants. The inter-trial interval was jittered between 2000 and 3000 ms in 250 ms steps.

Participants completed 20 practice trials using five faces not shown in the experimental blocks, followed by 5 blocks of 36 trials. Each block contained nine trials of each cue/face combination (2 cues × 2 faces), resulting in 36 trials per condition.

#### Mood Rating

The Positive and Negative Affect Schedule (PANAS, state version, [15]) was administered to measure potential mood changes induced by electrical stimulation. The PANAS is a commonly used 20-item self-report questionnaire, ten items to measure positive affect (PA) and ten items to measure negative affect (NA). PA represents emotions such as enthusiasm and alertness, and NA represents emotions such as subjective distress and un-pleasurable engagement. The PANAS has been found a reliable and valid measure of PA and NA [18].

#### EEG Apparatus

A Geodesic Sensor Net System (Electrical Geodesic, Inc., Eugene, OR) was used to record 128-channel EEG within an electrically and acoustically shielded room (sampling rate: 250 Hz; analog filter: 0.1 Hz; recording to average reference; impedances <45 kΩ). Responses were recorded using E-Prime Biological Add-ons for Net Station (Psychology Software Tools, Inc., Pittsburgh, PA).

#### tDCS

Direct electrical current was applied by a saline-soaked pair of surface sponge electrodes (35 cm^2^) and delivered by a battery-driven stimulator. To stimulate the left DLPFC, the anode electrode was positioned centered over F3 according to the 10–20 international system for electroencephalogram electrode placement. The cathode was placed over the contra lateral supraorbital area. This electrodes placement and method of DLPFC localization is in accordance with prior tDCS studies. A constant, direct current of 2 mA with 20 s of a ramp up was applied for 20 min. For sham stimulation, the electrodes were positioned similar as when administering tDCS stimulation; however, the current was ramped down after 20 seconds. This procedure is a reliable sham condition [1].

### Data reduction

#### Behavioral data

In total, the CECT consisted of 180 trials, resulting in 45 trials/target type (4 targets). Only correct responses were considered in analyses of RT. Overall, accuracy rates for all CECT trial types were high (88%–94%). Throughout the remainder of the manuscript, effects are described by the cue and then facial emotion (e.g., “opposite/happy” refers to the opposite cue followed by a happy face, which would require pressing the button labeled with “sad”).

#### Scalp ERP data

EEG data were analyzed using Netstation, and filtered offline with a 30 Hz low-pass filter (12dB/octave). Artifact detection was performed to identify artifacts: difference >55 µV between channels near the outer canthi, or one or more channels exceeding an amplitude of 200 µV were automatically rejected (moving average of 80 ms). Eye blinks were rejected when the difference was >140 µV, and eye movement were rejected when the difference was >55 µV. Subsequently, channels with corrupted signal were replaced using spatially weighted linear interpolations (Hjorth nearest neighbors algorithm). Next, stimulus-locked (−200 ms to 1000 ms) segments were extracted, only for those trials that were followed by a correct response. The tDCS and sham measurements did not differ in the mean number of segments available for ERP analyses [36.80±1.26 vs. 35.88±.93, *t*(25)  = .88, *p = *.38]. Finally, data were re-referenced to average and then were baseline-corrected (200 ms–0 ms).

After visual waveform inspection for maximal deflection locations, the N450 component was calculated by averaging the amplitudes between 450 and 630 ms following the presentation of the target. In line with prior research e.g., [19], [20], the topography of this potential was maximum over frontal-central electrodes distributed around the midline (average amplitude taken of electrodes Fz, EGI sensors 10, 16, 18).

### Statistical plan

Because of a significant gender difference (8M/17F), the basic statistical design always included gender as a between subjects factor to rule out the possible influence of gender on the effects. If this between subjects factor yielded no main effect and was not implied in any crucial interaction effect, this factor was left out in all further analyses.

To examine possible effects of tDCS on mood states, separate *Stimulation* (tDCS, sham) × *Time* (T_0_, T_1_, T_2_) ×*Gender* (male, female) mixed ANOVAs were performed on Negative Affect (NA) and Positive Affect (PA), as measured by the PANAS. Because one participant did not fill in the questionnaires, repeated measures analyses were performed on 24 participants.

For behavioral data, separate *Cue* (Opposite, Actual) × *Emotion* (Sad, Happy) × *Stimulation* (tDCS, sham) × *Gender* (male, female) mixed ANOVAs were performed on RT and accuracy scores of the CECT.

For analyses of the ERP data, a repeated measures ANOVA with *Cue* (Opposite, Actual) × *Emotion* (Sad, Happy) × *Stimulation* (tDCS, sham) × *Gender* (male, female) mixed ANOVA was performed on N450 amplitudes at frontocentral electrode sides.

Across analyses, significant ANOVA effects were followed-up using *t*-tests. Effect sizes for ANOVAs are reported in the form of partial eta squared (ηp^2^), where 0.05, 0.1, and 0.2 correspond to small, medium, and large effects, respectively. The significance level was set at an alpha level of .05.

## Results

Overall, tDCS was well tolerated with minimal side effects (transient headache, skin itching and redness).

### Effects on Mood

Gender did not show a significant main effect (*F*s<.15; *p*s>.7), nor was implied in any interaction effect (*F*s<1.7; *p*s>.2) with PA and NA as dependent variables, and was consequently removed from all further analyses. The *Stimulation* (tDCS, sham) × *Time* (T_0_, T_1_, T_2_) repeated measures ANOVA for PA revealed a significant main effect of *Time* [*F*(2, 22)  = 15.96, *p*<.0001, η_p_^2^ = .59]. The main and interaction effects with *Stimulation* were not significant (*F*s<1.06, *p*s>.36). Likewise, the *Stimulation* (tDCS, sham) × *Time* (T_0_, T_1_, T_2_) repeated measures ANOVA for NA revealed a significant main effect of *Time* [*F*(2, 22)  = 4.71, *p* = .02, η_p_^2^  = .30], but also no significant main or interaction effect with *Stimulation* (*F*s<.94, *p*s>.41). Paired t-tests revealed that both in the sham and tDCS condition, participants reported less PA and NA towards the end of the experiment: T1 to T0 (*p*<.05) and T2 to T1 (*p*<.05). For both PA and NA, the main effect and interaction with *Stimulation* did not reach significance, *F*s<1 & *p*s>.1. Therefore, changes in mood are not different between both stimulation conditions, and would therefore not confound with the effects of tDCS on cognitive control.

### Effects on Behavioral data

Gender did not show a significant main effect (*F*s*<*.53; *p*s<.50), nor was implied in any interaction effect involving *Cue* or *Emotion* (*F*s<.1; *p*s>.30) with RT or accuracy rates as dependent variables, and was consequently removed from all further analyses.

#### Reaction times

**Figure 2:**
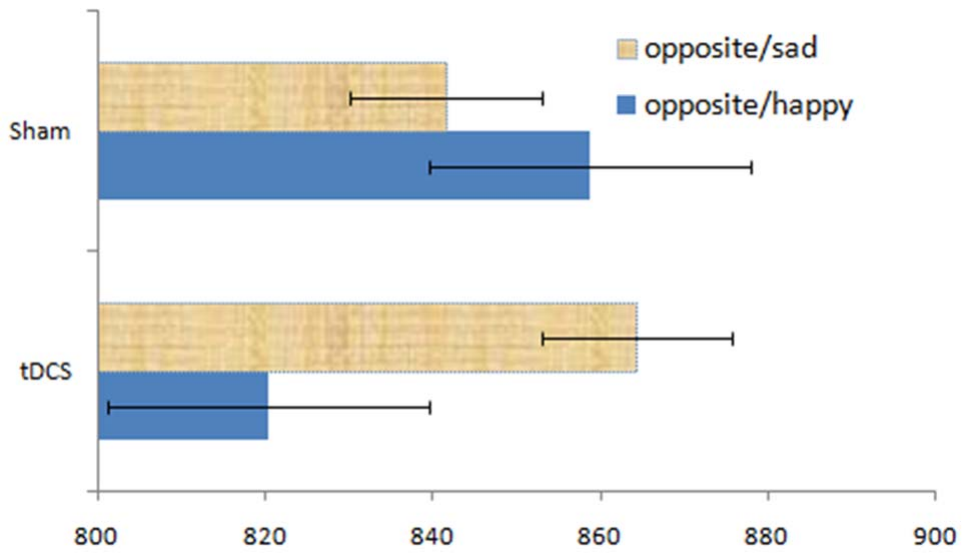
Mean RT for opposite trials (opposite/sad and opposite/happy) following tDCS and sham stimulation.

The *Cue* (Opposite, Actual) × *Emotion* (Sad, Happy) × *Stimulation* (tDCS, sham) repeated measures ANOVA for RT revealed a main effect of *Cue* [*F*(1, 24)  = 90.32, *p*<.0001, η_p_^2^ = .79], a main effect of *Emotion* [*F*(1, 24)  = 14.12, *p* = .001, η_p_^2^  = .37], an interaction between *Cue* and *Emotion* [*F*(1, 24)  = 20.05, *p*<.0001, η_p_^2^  = .46] and, most important, a significant three-way interaction [*F*(1, 24)  = 9.16, *p = *.006, η_p_^2^  = .28]. Paired t-tests revealed that following tDCS, RT to opposite/happy were significantly faster compared to opposite/sad trials, *t*(24)  = 2.43, *p = *.02, suggesting more cognitive control for positive material than for negative material. RT to opposite/sad and opposite/happy were not significantly different following sham stimulation, [*t*(24)  = 1.23, *p = *.23], see (Figure 2). Active or sham stimulation did not influence RT to actual/sad and actual/happy trials, for which emotion naming was faster for positive (*M_tDCS_* = 647.22; *M_Sham_* = 680.74) compared to negative (*M_tDCS_* = 726.02; *M_Sham_* = 766.58) information in both conditions, *t*s>4.23, *p*s<.001.

#### Accuracy rates

A *Cue* × *Emotion* × *Stimulation* ANOVA for accuracy scores revealed a main effect of Cue, [*F*(1, 24)  = 22.59, *p*<.0001, η_p_^2^ = .49] due to more errors on opposite compared to actual trials. No other effects were observed, suggesting no effects of tDCS on accuracy rates.

### Effects on Electrophysiological data

Gender did not show a significant main effect (*F = *.21; *p* = .65), nor was implied in any interaction effect involving *Cue* or *Emotion* (*F*>1.8; *p*<.20) with N450 amplitudes as dependent variables, and was consequently removed from all further analyses.

**Figure 3:**
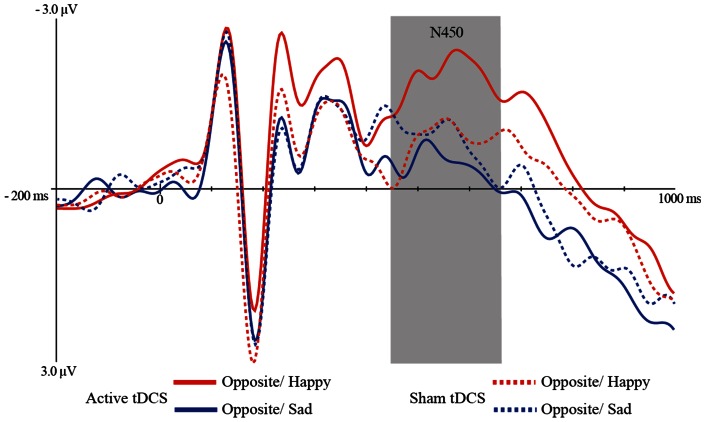
Target locked grandmean waveforms at electrode FCz for the opposite trials(opposite/sad and opposite/happy) following tDCS and sham stimulation.

The *Cue* (Opposite, Actual) × *Emotion* (Sad, Happy) × *Stimulation* (tDCS, sham) ANOVA yielded a main effect of *Emotion*, [*F*(1, 24)  = 11.39, *p* = .003, η_p_^2^ = .32], with more negative amplitudes during the processing of emotionally negative material, *p*s<.05. The *Cue* × *Emotion*, [*F*(1, 24)  = 6.36, *p* = .02, η_p_^2^ = .21] was significant with more negative N450 amplitudes for the opposite trials, which is in accordance with opposite trials that need more cognitive control. No other main or two-interaction effects were observed, *F*s<1.10, *p*s>.30. Most interestingly, a three-way interaction was found [*F*(1, 24)  = 4.53, *p* = .04, η_p_^2^ = .16]. Paired t-tests revealed that after tDCS, N450 amplitudes for opposite/happy trials (*M* = −2.68) were significantly more negative than opposite/sad trials (*M* = 1.14) (*t* = 2.69, *p* = .01). Opposite/happy trials were not significantly different from opposite/sad trials after sham stimulation, *p*>.50. These findings show that tDCS over the left DLPFC increased N450 amplitudes – a marker of cognitive control to overcome interference for positive relative to negative trials, see (Figure 3). Both in the sham and tDCS condition, amplitudes were significantly more negative for actual/happy compared to actual/sad trials, *t*s>.2.76, *p*s<.01.

## Discussion

The aim of the present study was to investigate the effects of a single sham-controlled, anodal tDCS session applied to the left DLPFC on cognitive control for affective stimuli, indexed by RT and ERPs during the CECT. Importantly, and in line with previous non-invasive brain stimulation studies [21], [22], active versus sham tDCS did not differentially influence mood states. Therefore, it is unlikely that changes in mood have influenced our outcome of cognitive control on emotion. Moreover, gender did not influence the effects of anodal tDCS on cognitive control for emotional information. This is important because gender has been found to influence ERP components in response to emotional stimuli e.g., [23].

Behavioral data show a valence specific effect on cognitive control following active tDCS – i.e. participants were found to respond faster when inhibiting a habitual response to positive (opposite/happy trials) relative to negative (opposite/sad trials) affective material. These findings suggest that participants could specifically enhance cognitive control more for positive relative to negative information following tDCS. In line with these behavioral results, electrophysiological data revealed more negative N450 amplitudes for opposite/happy compared to opposite/sad stimuli, only for active (but not sham) tDCS. These enhanced N450 amplitudes involve the recruitment of more cognitive control to overcome interference from conflicting mental representations [11]. Source localization analyses have identified regions within the anterior cingulate cortex (ACC) as the potential generator of the N450 component [24]. A core function of the ACC is the monitoring of conflict, namely to increase attentional control to overcome an automatic response in the presence of a distracter. It is well known that if a conflict between competing (emotional) representations is present, a conflict monitor localized in the ACC will be activated, which will in turn signal to the DLPFC in order to engage control and improve performance [8]. tDCS seems to have modulated this ACC-DLPFC neural circuitry, reflected in more negative polarities for the N450 component and faster RT, but specifically for positive affective stimuli.

These emotion specific effects were only observed for cognitive control operations and not for the naming of these stimuli (i.e., RT following the cues “actual”), rejecting the possibility that tDCS would lead to a non-specific increase focal brain activity (e.g., random noise which would lead to facilitated identification or recognition of specific valences). These observations are in line with the hypothesis that tDCS specifically enhances the signal-to-noise ratio of cognitive control for emotional processing [25]. In other words, tDCS over the left DLPFC might functionally activate a neural network that is specifically engaged during tasks that require cognitive control, namely the ACC-DLPFC neural circuitry, and thereby reducing the threshold to detect associated behavioral outcome.

These valence specific observations, namely increased cognitive control for positive (versus negative) material after neuromodulation of the left DLPFC, seem in agreement with the Valence Theory of side-lateralized activity of the prefrontal cortex in emotional processing [26], [27]. This theory states that the preferential processing of positive and negative stimuli would be lateralized towards the left and right prefrontal cortex, respectively. Possibly, anodal tDCS of the left DLPFC enhanced activation in the left hemisphere, leading to preferential cognitive control for positive information. Although side-dominant activation can be observed, tDCS has wide spreading effects to subcortical but also contra-hemispheric regions [28], [29], [30]. Therefore, it is likely that, following a single tDCS session over the left DLPFC, the relatively more dominant left (versus the right) prefrontal cortex activation might have caused (as seen in this study) the enhancement of cognitive control specifically for positive material and reduced cognitive control for negative affective stimuli. This might explain why we did not observe absolute differences between tDCS and sham stimulation in the processing of affective material, but instead observed a relative increase in cognitive control for positive compared to negative information.

Importantly, more research is needed to support the abovementioned hypothesis that anodal tDCS of the DLPFC would generate results that are in line with the Valence Theory of side-lateralized activity of the prefrontal cortex in emotional processing [26], [27]. In this study, we only investigated the effects of anodal tDCS of the left DLPFC, which is the mostly used montage for looking at the effects of tDCS on cognitive control. Future research should however investigate the effects of tDCS of a sham controlled left/right DLPFC electrode montage and, perhaps, combine it with neuroimaging techniques. If tDCS induces effects on cognitive control that are in line with the Valence Theory of side-lateralized activity [26], [27], then (1) anodal stimulation of the right areas would selectively enhance cognitive control for negative stimuli, and (2) anodal stimulation of the homologue left areas would selectively enhance cognitive control for positive stimuli. Neuroimaging correlates would make it possible to investigate side lateralized neural activation during cognitive control for emotional information following a left/right DLPFC electrode montage.

In conclusion, this study suggests that a single sham-controlled anodal tDCS session over the left DLPFC causally and specifically enhances cognitive control for positive relative to negative information, indexed by more negative N450 amplitudes and faster RT when inhibiting a habitual response towards positive relative to negative material. Although more research is needed, our results are in line with the lateralized hemisphere theory of affect processing [26] and also expand the knowledge of the mechanisms of action of tDCS by showing its role in cognitive control for emotional information.
